# Enhanced wedelolactone content in *in vitro*-raised genetically uniform *Wedelia chinensis* under the influence of CuSO_4_

**DOI:** 10.3389/fpls.2023.1281445

**Published:** 2023-12-19

**Authors:** Ravi Kant Swami, Shwetanjali Nimker, Alka Narula, Humaira Farooqi

**Affiliations:** ^1^Department of Biotechnology, School of Chemical and Life Sciences, Jamia Hamdard, New Delhi, India; ^2^BD-JH FACS Academy, New Delhi, India

**Keywords:** conservation, flow cytometry, HPTLC, wedelolactone, quantification

## Abstract

In the present study, we addressed the imperative for potent anticancer agents through *Wedelia chinensis*, a medicinal plant abundant in the robust antihepatotoxic and antitumor compound wedelolactone. Hindrances in conventional propagation methods due to cross-pollination and habitat degradation prompted us to pioneer *in vitro* rapid multiplication using plant tissue culture. Optimal outcomes were attained employing Murashige and Skoog (MS) medium supplemented with Indole-3-butyric acid (IBA) (0.5 mg/L) and Kinetin (KN) (5.0 mg/L), yielding 97.67% shoot regeneration and 81.67% rooting from nodal explants. Transplanted plantlets exhibited a 92% survival rate. We established a wedelolactone extraction protocol using toluene:ethyl acetate:formic acid (5:4:1) for High-performance thin-layer chromatography (HPTLC) analysis, trailblazing wedelolactone quantification and 2C DNA analysis in *W. chinensis *via flow cytometry. Experiments under heavy metal stress with CuSO_4_ unveiled physiological responses, with peak wedelolactone content [193.90 μg/g dry weight (dw)] *in vitro* at 75 μM CuSO_4_, surpassing *in vivo* levels (89.95 μg/g dw) by 116%. By pioneering successful *in vitro* rapid multiplication and enhanced wedelolactone content, we bridge a critical gap in the conservation and production of this medicinal plant. Our findings not only offer a sustainable means of propagation but also present a viable strategy for elevating the yield of potent bioactive molecules like wedelolactone, holding immense promise for the development of novel therapeutic interventions and addressing the pressing healthcare challenges of our time.

## 1 Introduction

*Wedelia chinensis*, commonly known as Pilabhangra, is a well-known medicinal plant of Asteraceae (Sunflower family). It is found in India, Sri Lanka, China, Indonesia, Japan, Philippines, and Malaysia (Pratap et al., 2012). *W. chinensis* is used traditionally as a remedy for liver disorders (Mishra et al., 2009; Naveen and Sree, 2012), osteoporosis of the knee (Nomani et al., 2013), dermatological disorders (Koul et al., 2012), inflammation (Manjamalai and Grace, 2012), multiple sclerosis (Nithin Manohar et al., 2017), rheumatic fever, and most importantly cancer (Lin et al., 2007; Tsai et al., 2009; Manjamalai and Grace, 2013). It contains wedelolactone (coumestan derivative) possessing potent antihepatotoxic and antitumor activity against various types of cancer cell lines like prostate (Lin et al., 2007; Tsai et al., 2009), lung (Manjamalai and Grace, 2013), and breast (Benes et al., 2011; Xu et al., 2014; Hsieh et al., 2015). The increasing problem of cancer in today’s era also needs more anticancerous and effective drugs. To circumvent this problem, *W. chinensis* could be a good source.

This plant can be grown with seeds and vegetative stem cuttings. However, because of cross-pollination, the germinated seedlings are not true to type, and multiplication via this way is not trustworthy because of low viability (Agarwala et al., 2010). Moreover, indiscriminate collection, habitat loss, urbanization, and increasing demand for wedelolactone may lead to the extinction of this plant from its natural environment. Previously, *in vitro* mass multiplication through different explants, such as nodal segments (Bhuyan et al., 2000; Agarwala et al., 2010; Rahman and Bhadra, 2011), axillary buds (Martin et al., 2003; Islam et al., 2009), shoot tips (Islam et al., 2009; Agarwala et al., 2010), and young leaf explant (Tsai and To, 2021) has been reported. However, no effective transplantation and hardening procedures were reported. Furthermore, no such reports of extraction and quantification of wedelolactone are reported in the literature. To overcome this situation, in the present report, *in vitro* rapid multiplication and transplantation for the conservation of the germplasm of *W. chinensis* and commercial production of wedelolactone from this important medicinal plant have been demonstrated.

In addition, plant growth regulators used in tissue culture not only provide high production of biomass but also contribute to the enhancement of secondary metabolites. Auxin and cytokinin fortified into the culture medium have a marked influence on the production of secondary metabolites by increasing cell metabolism (Al-Qudah et al., 2011). In *Teucrium polium*, the amount of β-caryophyllene was reported high when grown *in vitro* on Murashige and Skoog (MS) medium supplemented with 6-Benzylaminopurine (BA) and Naphthaleneacetic acid (NAA) than that grown on hormone-free MS medium (Al-Qudah et al., 2011). Similarly in *Stevia rebaudiana*, the combination of BA with Gibberellic acid (GA_3_) and Indole-3-Acetic Acid (IAA) strongly affects the accumulation of secondary metabolites and results in the enhancement in the yield of bioactive polyphenolics (Radić et al., 2016).

Regeneration of plants through plant tissue culture is the best technique to produce true-to-type plants (Ali et al., 2017). However, the genetic stability of *in vitro*-grown plants can be altered by mutation and error in cell division, which results in the formation of genetically and genomically variant cells (Naing et al., 2014). Moreover, genetic alterations may be affected by the age of the culture (Samarina et al., 2019), the composition of the culture medium, and specifically the different combinations and concentrations of plant growth regulators (Gantait and Vahedi, 2015). Furthermore, the influence of genetic disturbance can also result in a change in ploidy level, and elevated ploidy among regenerants plays a vital role in changing the phenotypic characteristics and formation of genetically manipulated plants (Jing et al., 2016). All of these parameters can diminish the commercial value of *in vitro*-raised plants (Oh et al., 2007). Thus, it is necessary to access genetic stability to preserve the desirable features in *in vitro*-raised plants. The investigation of the genetic stability of *W. chinensis* was done using Random Amplified Polymorphic DNA (RAPD) markers (Kundu et al., 2017). Despite the various advantages, RAPD has a drawback of reproducibility. Flow cytometric technique these days proved to be a key to reveal not only the genetic fidelity but also helping to check the ploidy level of regenerated plants and genome size (Malik et al., 2020).

Pharmaceutically important secondary metabolites are produced in response to various biotic and abiotic stresses and help the plant survive under unfavorable conditions. The heavy metal stress influences the production of secondary metabolites by triggering multiple signaling pathways. The accumulation of polyamines is also reported in numerous plant species under stress conditions (Jankovska-Bortkevič et al., 2022).

This present study aimed to develop an improved and efficient *in vitro* mass multiplication protocol and to check the genetic stability and genome size determination of *in vitro*-grown plantlets using flow cytometry. The present study also inspected the quantification of wedelolactone content in *in vitro*-raised and field-grown plants as well as under the influence of CuSO_4_ stress plantlets through HPTLC.

## 2 Materials and methods

### 2.1 Plant material and surface sterilization

Nodal segments of *W. chinensis* were collected from the Herbal Garden of Jamia Hamdard, New Delhi. The explants were cut into 3–4-cm single nodes and washed three times with running tap water. The washed explants were sequentially surface-sterilized with 0.2% cetrimide (5 min), 0.25% streptomycin sulfate (10 min), 0.5% Bavistin (7 min), 0.1% mercuric chloride (5 min), and finally with 70% alcohol for 1 min. The sterilized nodal segments were finally transferred to sterile double-distilled water (ddH_2_O). The sterile explants were trimmed at both ends before the inoculation.

### 2.2 Medium preparation and culture conditions

For regeneration through nodal segments, MS medium (Murashige and Skoog, 1962) with different concentrations and combinations of auxin (IBA/NAA) with cytokinin (BA/KN) was prepared. MS medium devoid of growth regulators served as control. Each medium was supplemented with 3% (w/v) sucrose. For the semisolid medium, 0.63% agar was used. However, for better root proliferation, a liquid MS medium supplemented with IBA (0.5 mg/L) and KN (5.0 mg/L) was prepared. For the support of plantlets in liquid medium, two layers of glass beads were formed in a 100-mL flask. The pH of both the semisolid and the liquid MS medium was adjusted to 5.69 using 1 N NaOH/1 N HCl and subsequently poured into 100-mL flasks. The medium was autoclaved at 121°C at 15 psi for 15 min. The efficiency of the explant was analyzed based on parameters like percent regeneration, number, and length of shoots and roots. *In vitro*-grown plantlets were subcultured every fourth week. The cultures were maintained in the culture room at 25°C ± 2°C with 50% ± 5% relative humidity and 16/8 h photoperiod.

### 2.3 *Ex vitro* acclimatization and transplantation in the field

Plantlets with well-developed thick roots were washed with sterile ddH_2_O and transferred to pots containing Soilrite and soil (1:1) and covered with transparent polyethylene bags with small holes. The plants were irrigated with half strength of MS liquid basal medium every 7 days. These pots were transferred and maintained in growth chambers at 25°C ± 2°C for 16 h of illumination. After 1 month of acclimatization, covers were withdrawn and the plants were transferred to the greenhouse for adaptation, irrigated with tap water, and finally transferred to the field.

### 2.4 Phytochemical screening

#### 2.4.1 Extraction of wedelolactone

*In vivo*, *in vitro*-grown plants raised on different media and transplanted plantlets were harvested for wedelolactone estimation. The samples were oven-dried at 50°C and ground to a fine powder with the help of mortar and pestle. Dried powder (5 g) was dissolved in methanol and kept in a shaker overnight. Thereafter, sonication of extract was done for 30 min at 60°C. Thereafter, the extract was boiled in a water bath for 10 min and filtered through a Whatman filter paper (No. 42). The filtrate was kept in a water bath for solvent evaporation, and the residue obtained was kept in the oven overnight. The dried residue was again suspended in ddH_2_O and partitioned with ethyl acetate (three times). Ethyl acetate fractions were collected and evaporated to dryness in a water bath. The dried residue was dissolved in 5 mL methanol [High-performance liquid chromatography (HPLC) grade] and used for HPTLC analysis.

#### 2.4.2 Determination of the solvent system and absorption maxima of wedelolactone

A stock solution of standard (wedelolactone) was prepared by dissolving 1.0 mg standard in 1.0 mL of HPLC-grade methanol, and further dilutions were made. Different combinations of organic polar (ethyl acetate, formic acid, methanol, acetone, and acetic acid) and nonpolar (toluene, chloroform, and diethyl ether) solvents were used in the Thin-layer chromatography (TLC) for the determination of the solvent system. The absorption maxima for the standard wedelolactone were determined using both UV spectroscopy and HPTLC.

#### 2.4.3 HPTLC analysis

HPTLC of the methanolic extract of different samples was performed using CAMAG, Switzerland. Plant extracts were applied with a 100-μL syringe on precoated silica gel 60 HPTLC plates (10 cm × 10 cm) after activation at 120°C with a band length of 6 mm and a track separation of 10 mm using Linomat V application device. The chromatograph was developed in a twin trough chamber using a solvent system and scanned in scanner III at 366 nm using UV lamp in absorption mode.

The wedelolactone was detected based on the R_f_ value of standard wedelolactone (99.9%) (Sigma). For quantitative analysis, peak areas were used to calculate the amount of wedelolactone present in the tissues, and these were compared with the standard. The standard sample was used to construct a calibration graph by plotting the peak area vs. the amount of wedelolactone.


Y = 15.663  +  9.168 *X

Here, Y = Area of peak, X = Concentration of wedelolactone.

The concentration of wedelolactone was calculated from the above formula. The samples were analyzed in triplicate.

### 2.5 Flow cytometry-based acquisition and analysis of *in vitro* and *in vivo* samples

All of the flow cytometric analyses were carried out by using a BDFACS Verse flow cytometer (Becton Dickinson, 8-color configuration). Firstly, BD DNA QC beads (Cat. No.-349523) were run containing the chicken erythrocyte nuclei (CEN) and calf thymocyte nuclei (CTN) fluorescent particles to set the instrument photomultiplier tube (PMT) voltages, amplifier gains, and providing information regarding instrument linearity and resolution.

Unstained blood samples were used to gate the main population within the side scatter area linear scale (SSC-A-lin) vs. the forward scatter height area scale (FSC-A-lin) and to determine the correct voltage and gain settings. Propidium iodide area linear (PI-A) vs. propidium iodide width linear (PI-W) plots were made to distinguish the singlet cells and to simultaneously adjust the gain settings of the cytometer to position the 2C peak at channel 100 in the histogram plot (PI-A vs. count). The statistics of the positive population are determined by the BDFACS Suite software from a total of 10,000 cells. For the internal reference standard, *Pongamia pinnata* was taken (2C = 2.51 pg DNA) (Choudhury et al., 2014).

Determination of nuclear DNA content of field-grown, *in vitro*-regenerated 4-month-old plantlet and 6-month-old transplanted regenerants of *W. chinensis* was done. Here, 20 mg of young leaf segments of *W. chinensis* and *P. pinnata* were finely chopped using a sharp razor blade in 0.5 mL Galbraith buffer of pH 7 [45 mM MgCl_2_, 20 mM MOPS, 30 mM sodiucitrate, 0.1% (v/v) Triton X-100] on ice. The nuclear suspensions were filtered through a 40-μm nylon mesh cell strainer (HIMEDIA) followed by the addition of 0.5 mL of stock solution of PI/RNase staining buffer (BD Pharmingen™, BD Biosciences, USA). The suspension was kept for half an hour of incubation with continuous vortex in every 5-min interval before being analyzed in the flow cytometer. The samples were processed by following the protocol (Doležel et al., 2007). The genetic stability of the nuclear suspension was examined by comparing the median of 2C peak of histogram (PI-A vs. count) of plants regenerated through tissue culture (4-month-old plantlet and 6-month-old transplanted regenerant) and field-grown *W. chinensis*. The 2C DNA content of *W. chinensis* was determined using the below formula:


$$2\text{C DNA content of}\;W.\;\text{chinensis}=\;2.51\times \;\frac{{\text{Median position of G}}_{0}/{\text{G}}_{1}\text{ peak of }W.\text{chinensis}}{{\text{Median position of G}}_{0}/{\text{G}}_{1}\text{ peak of }P.\text{pinnata}}$$

### 2.6 CuSO_4_ stress tolerance in regenerants

For the cultivation of metal-tolerant plants, shoots regenerated from the *in vitro*-raised plantlets were cut into 4–5-cm-long pieces and transferred to MS medium supplemented with IBA and KN (0.5 + 5.0 mg/L) additionally supplemented with various levels (25, 50, 75, 100, and 125 μm) of CuSO_4_. One set of cultures maintained on a medium without any heavy metal served as a control. Shoot fresh weight (fw), shoot dry weight (dw), protein content, proline accumulation, chlorophyll, carotenoid content, and wedelolactone yield were monitored after 12 weeks.

#### 2.6.1 Shoot fresh and dry weight

Shoots grown on different CuSO_4_ concentrations and control were harvested after 12 weeks, and fw was done. The shoots were kept in an oven at 50°C, and the shoot dw was calculated.

#### 2.6.2 Protein estimation

Soluble protein content was estimated by following the method of Bradford (1976). Fresh tissue (100 mg) was homogenized in 1 mL of 0.1 M phosphate buffer with the help of a mortar and pestle, precooled, and kept in ice. The homogenate was centrifuged at 6,000 rpm for 10 min at 4°C. An equal amount of chilled 10% Trichloroacetic acid (TCA) was added to the supernatant and incubated for 20 min, which was again centrifuged at 3,300 rpm for 10 min.

The supernatant was discarded, and the pellet left was washed with acetone. It was then dissolved in 1 mL of 0.1 N NaOH. Here, 0.1 mL from the solution was kept in a centrifuge tube, and 0.5 mL of Bradford’s reagent was added, vortex-mixed, and kept for 10 min for optimal color development. The absorbance was then recorded at 595 nm on a UV-Vis spectrophotometer (Lambda Bio 20, Perkin Elmer). The soluble protein concentration was quantified with the help of a standard curve prepared from bovine serum albumin. The amount of protein was expressed as mg/g fw.

#### 2.6.3 Proline content

Proline content in the leaves was determined by the method of Bates et al. (1973). For proline determination, the fully expanded leaves were detached from the plants after the CuSO_4_ treatment. Here, 1.0 g of leaf samples were homogenized in 10 mL of sulfosalicylic acid (3%) using mortar and pestle followed by centrifugation at 10,000 rpm for 10 min at room temperature. After centrifugation, 2 mL of supernatant was taken in a test tube, and 2 mL of glacial acetic acid and 2 mL of ninhydrin reagent were added to it. The reaction mixture was boiled in a water bath at 100°C for 30 min. The reaction was stopped by keeping it at a low temperature. After that, 4 mL of toluene was added in the mixture and vortexed. The absorbance was recorded at 520 nm in a spectrophotometer against toluene as blank. The concentration of proline was estimated by referring to a standard curve of proline.

#### 2.6.4 Chlorophyll and carotenoid content

The chlorophyll content of the biological sample was determined by the Hiscox and Israelstam (1979) method. For this, 0.1-g plant leaves were weighed and incubated in 10 mL DMSO for 1 h at 65°C, and the Optical Density (OD) of the supernatent was measured at 480 nm, 510 nm, 645 nm, and 663 nm. Chlorophyll a, chlorophyll b, total chlorophyll, and carotenoid (mg/g fw) contents were then calculated from the following formula:


$$\text{Chlorophyll a }\left(\text{mg}/\text{g fw}\right)\;\;\;\;\;\;\;\;=\;\frac{\left[12.3\;\left(\text{OD }663\right)\;+\;0.68\;\left(\text{OD }645\right)\right]\text{ X V}}{\left(\text{d X }1000\text{ X W}\right)}$$

$$\text{Chlorophyll b }\left(\text{mg}/\text{g fw}\right)\;=\;\frac{\left[19.3\;\left(\text{OD }645\right)\;-\;3.6\;\left(\text{OD }663\right)\right]\text{ X V}}{(\text{d X }1000\text{ X W)}}$$

$$\begin{array}{l} \text{Total Chlorophyll }\left(\text{mg}/\text{g fw}\right)\;=\;\frac{\left[20.2\;\left(\text{OD }645\right)\;+\;8.02\;\left(\text{OD }663\right)\right]\text{ X V}}{\left(\text{d X }1000\text{ X W}\right)}\; \end{array}$$

$$\text{Carotenoid}\left(\text{mg}/\text{g fw}\right)=\;\frac{\left[7.6\;\left(\text{OD }645\right)\;+\;1.49\;\left(\text{OD }663\right)\right]\text{ X V}}{\left(\text{d X }1000\text{ X W}\right)}$$

where d = Distance traveled by the light path.

W = Weight of the leaf material taken.

V = Volume of the extract.

#### 2.6.5 Wedelolactone quantification

Shoots grown on regeneration medium along with different CuSO_4_ concentrations and control were harvested after 12 weeks. The extraction and quantification were done by the above mentioned protocol.

### 2.7 Statistical analysis

The data on the effect of growth hormones on direct regeneration from nodal segments and proliferation along with the quantified wedelolactone using HPTLC were expressed as mean ± standard error. Each tissue culture experiment was conducted in replicates of 8 and was repeated thrice. However, in HPTLC, three replicates were used from three sets of the same experiment. The flow cytometry analysis was piloted by selecting plants randomly from *in vivo*- and *in vitro*-grown plants on BDFACS Verse. The mean values of each experiment were separated on SPSS software using Duncan’s multiple range test (DMRT) at a significance of p = 0.05 (Duncan, 1955). Determination of the median and CV of the gated single cell population was done using BDFACS Suite software.

## 3 Results

### 3.1 Direct regeneration and proliferation

**Figure 1:**
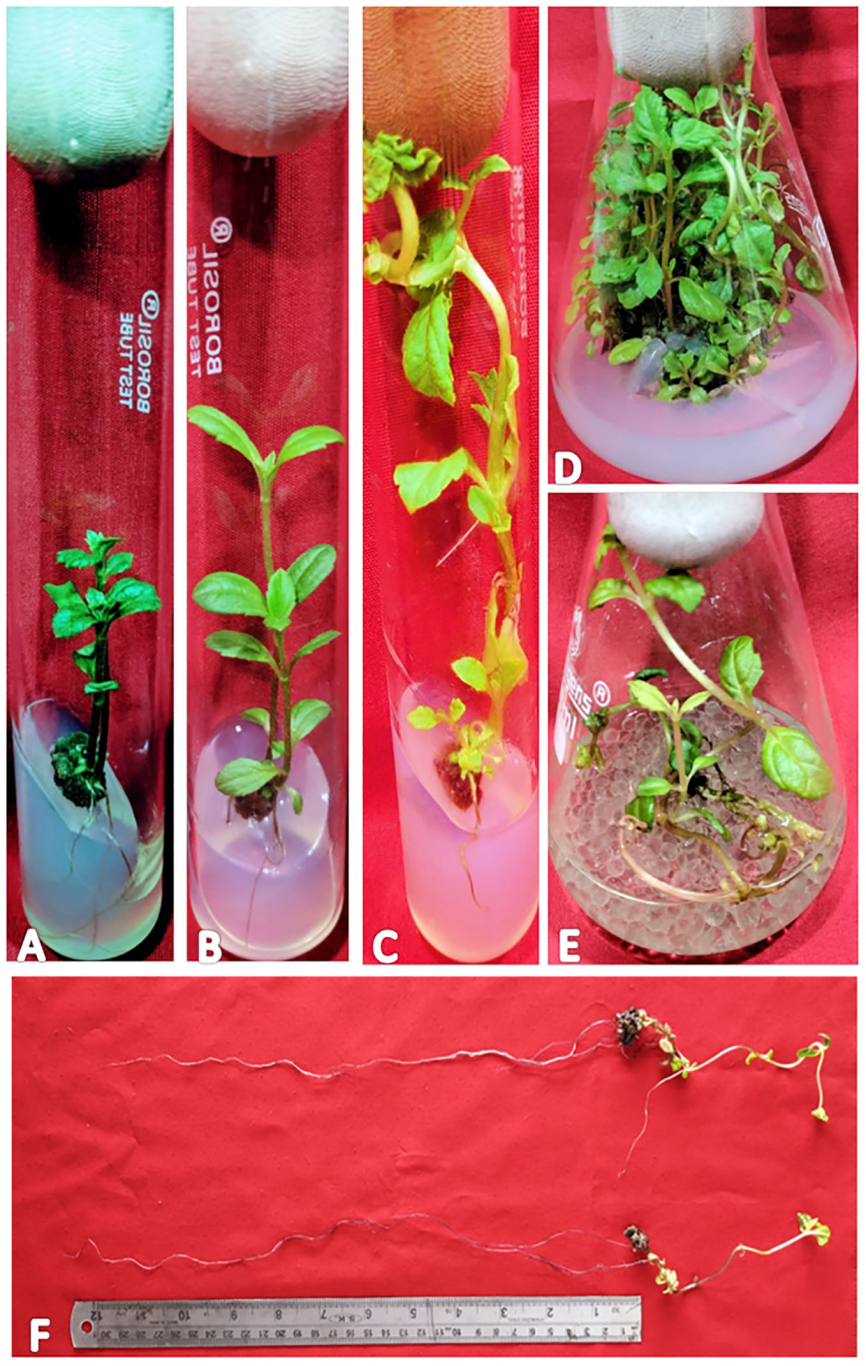
Different stages of regeneration in *W. chinensis*, Nodal segments grown on MS medium augmented with IBA (0.5 mg/l) and KN (5.0 mg/l) - **(A)**: 4-week-old culture, **(B)**: 8-week-old culture, **(C)**: 12-week-old culture, **(D)**: Shoot multiplication in 16-week-old culture, **(E)**: Root proliferation in MS (Liquid medium) containing IBA (0.5 mg/l) and KN (5.0 mg/l), **(F)**: 7-fold increase in root length on liquid MS medium supplemented with IBA (0.5 mg/l) and KN (5.0 mg/l). Note the growth of roots obtained in **(E)**.

**Figure 2:**
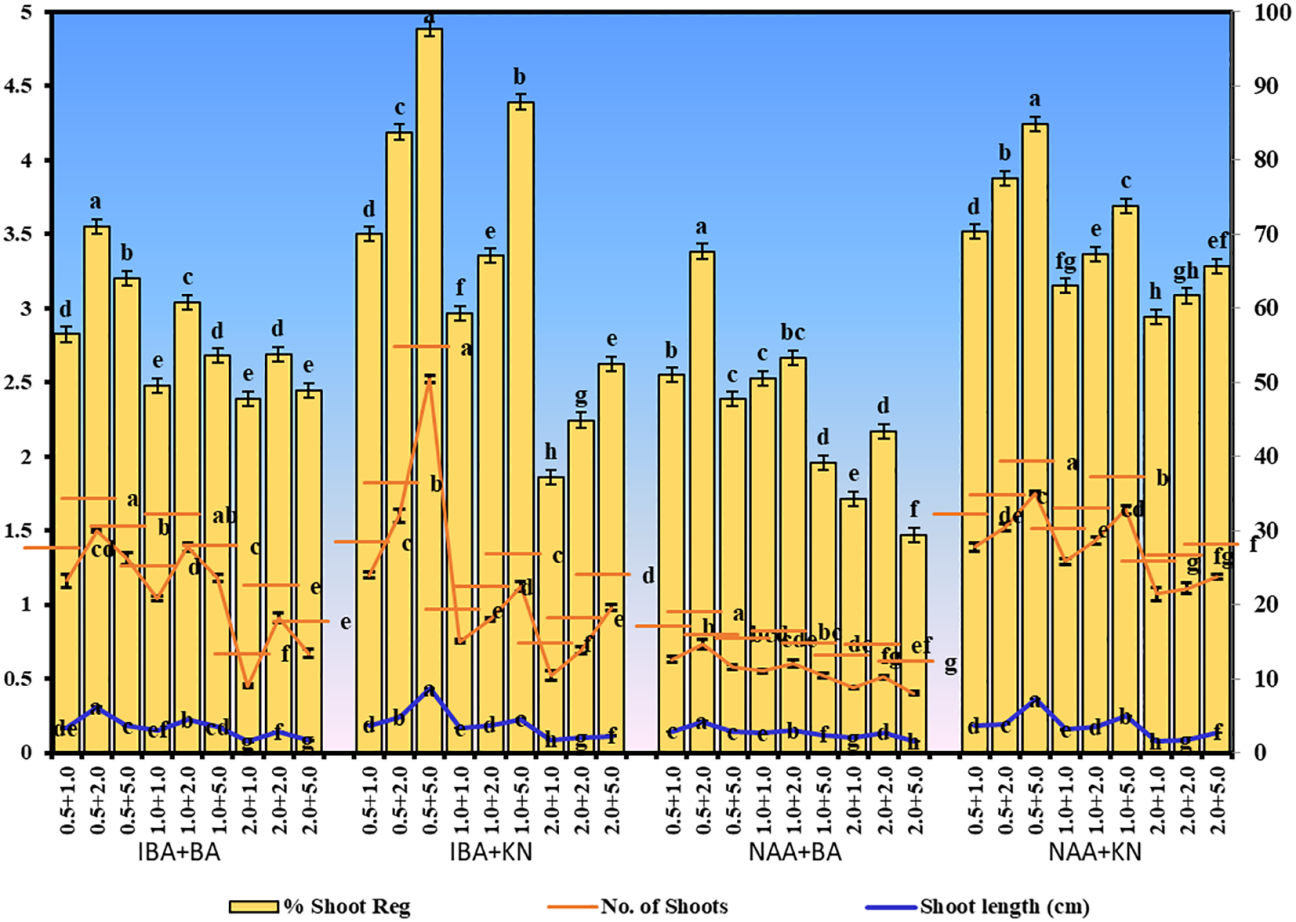
Shoot regeneration in *W. chinensis* through nodal segments after 12 weeks in the presence of different hormone combinations. Different lowercase letters are representing significance at p = 0.05 according to Duncan’s multiple range test.

In the present study, a lower concentration of auxin along with a higher concentration of cytokinin is better in terms of regeneration efficiency of the nodal segment. Among the phytohormones used, the combination of IBA and KN was found best for mass multiplication of this plant from nodes under *in vitro* conditions. Shoot regeneration efficiency of nodal explants in the presence of MS medium containing auxins (IBA or NAA: 0.5 mg/L, 1.0 mg/L, and 2.0 mg/L) along with higher concentrations of cytokinins (BA or KN: 1.0 mg/L, 2.0 mg/L, and 5.0 mg/L) has been tested. However, nodes inoculated on MS basal medium failed to regenerate even after 12 weeks. Among different concentrations of IBA with BA, 71.07% shoot regeneration with an average of 1.5 shoots of 6.16 cm per explant was obtained in the presence of MS containing IBA (0.5 mg/L) and BA (2.0 mg/L). On increasing the concentrations of either IBA or BA in the medium, the regeneration percentage declined. Furthermore, the use of KN as a cytokinin gave better regeneration efficiency. The IBA along with KN combinations was found to be best for shoot regeneration. Maximum 97.67% explants regenerated after 12 weeks in the presence of MS medium supplemented with 0.5 mg/L of IBA along with 5.0 mg/L of KN. This combination produced an average of 2.52 shoots of 8.65 cm within 12 weeks (**Figures 1A–C**). **Figure 1D** shows the maximum shoot multiplication on this medium. Furthermore, MS medium containing NAA and BA was found to be the least effective for shoot regeneration among all combinations used. Shoot regeneration efficiency was 67.67% with 0.73 shoots of 4.23 cm obtained in the presence of MS medium enriched with NAA (0.5 mg/L) along with BA (2.0 mg/L). However, NAA in combination with KN has better regeneration efficacy as compared with BA. An average of 84.90% shoot regenerated with 1.75 shoots/explant with an average length of 7.26 cm attained in the presence of MS medium with higher cytokinin (KN at 5.0 mg/L) and lower auxin (NAA at 0.5 mg/L) (**Figure 2**). All combinations of growth regulators resulted in callus formation at the basal cut end of the node. The detailed results are given in **Table 1**.

**Table 1:** Effect of plant growth regulators on shoot regeneration in *W. chinensis* through nodal segments after 12 weeks.

MS semisolid + PGR (mg/l)	Percent shoot regeneration	Average no. of shoots	Average length of shoots (in cm)
IBA + BA			
0.5+1.0	56.47 ± 1.07^d^	1.16 ± 0.046^cd^	3.30 ± 0.1465^de^
0.5+2.0	71.07 ± 0.64^a^	1.50 ± 0.012^a^	6.16 ± 0.0455^a^
0.5+5.0	64.07 ± 0.61^b^	1.31 ± 0.038^b^	3.70 ± 0.0510^c^
1.0+1.0	49.60 ± 0.70^e^	1.04 ± 0.018^d^	3.08 ± 0.0615^ef^
1.0+2.0	60.77 ± 1.06^c^	1.39 ± 0.029^ab^	4.45 ± 0.0450^b^
1.0+5.0	53.57 ± 0.71^d^	1.18 ± 0.022^c^	3.52 ± 0.0386^cd^
2.0+1.0	47.77 ± 0.79^e^	0.45 ± 0.012^f^	1.49 ± 0.0438^g^
2.0+2.0	53.80 ± 0.85^d^	0.91 ± 0.031^e^	2.78 ± 0.0450^f^
2.0+5.0	48.90 ± 0.97^e^	0.67 ± 0.026^e^	1.66 ± 0.0309^g^
IBA+KN			
0.5+1.0	70.00 ± 0.82^d^	1.20 ± 0.0211^c^	3.72 ± 0.0902^d^
0.5+2.0	83.78 ± 0.63^c^	1.60 ± 0.0471^b^	4.73 ± 0.0450^b^
0.5+5.0	97.67 ± 0.54^a^	2.52 ± 0.0237^a^	8.65 ± 0.0284^a^
1.0+1.0	59.33 ± 0.27^f^	0.75 ± 0.0109^e^	3.36 ± 0.0438^e^
1.0+2.0	67.10 ± 0.40^e^	0.90 ± 0.0127^d^	3.71 ± 0.0662^d^
1.0+5.0	87.85 ± 0.84^b^	1.12 ± 0.0318^c^	4.55 ± 0.0378^c^
2.0+1.0	37.17 ± 0.59^h^	0.52 ± 0.0314^f^	1.77 ± 0.0196^h^
2.0+2.0	44.89 ± 0.40^g^	0.69 ± 0.0227^e^	1.97 ± 0.0260^g^
2.0+5.0	52.50 ± 0.24^e^	0.98 ± 0.0191^d^	2.26 ± 0.0287^f^
NAA+BA			
0.5+1.0	50.97 ± 0.495^b^	0.63 ± 0.0205^b^	2.91 ± 0.02126^c^
0.5+2.0	67.67 ± 0.544^a^	0.73 ± 0.0339^a^	4.23 ± 0.02373^a^
0.5+5.0	47.70 ± 0.759^c^	0.58 ± 0.0146^bcd^	2.89 ± 0.01515^c^
1.0+1.0	50.57 ± 0.709^c^	0.55 ± 0.0094^cde^	2.62 ± 0.02842^e^
1.0+2.0	53.33 ± 0.720^bc^	0.60 ± 0.0255^bc^	3.02 ± 0.01886^b^
1.0+5.0	39.17 ± 0.936^d^	0.52 ± 0.0170^de^	2.42 ± 0.01656^f^
2.0+1.0	34.30 ± 0.648^e^	0.44 ± 0.0054^fg^	2.01 ± 0.02842^g^
2.0+2.0	43.37 ± 0.341^d^	0.51 ± 0.0119^ef^	2.75 ± 0.00943^d^
2.0+5.0	29.37 ± 0.746^f^	0.40 ± 0.0125^g^	1.54 ± 0.02944^h^
NAA+KN			
0.5+1.0	70.33 ± 0.95^d^	1.39 ± 0.0283^de^	3.62 ± 0.0288^d^
0.5+2.0	77.53 ± 0.83^b^	1.52 ± 0.0223^c^	3.81 ± 0.0497^c^
0.5+5.0	84.90 ± 0.59^a^	1.75 ± 0.0120^a^	7.26 ± 0.0213^a^
1.0+1.0	63.03 ± 0.86^fg^	1.29 ± 0.0182^e^	3.11 ± 0.0438^e^
1.0+2.0	67.33 ± 0.91^e^	1.43 ± 0.0223^cd^	3.54 ± 0.0381^d^
1.0+5.0	73.83 ± 0.58^c^	1.64 ± 0.0299^b^	4.94 ± 0.0331^b^
2.0+1.0	58.87 ± 0.85^h^	1.07 ± 0.0410^g^	1.52 ± 0.0196^h^
2.0+2.0	61.67 ± 0.76^gh^	1.11 ± 0.0334^fg^	1.72 ± 0.0260^g^
2.0+5.0	65.70 ± 0.60^ef^	1.19 ± 0.0170^f^	2.74 ± 0.0163^f^

Data in each column represents mean ± SE followed by different letters that are significantly different at p = 0.05 according to Duncan’s multiple range test.

### 3.2 Root induction and acclimatization

**Figure 3:**
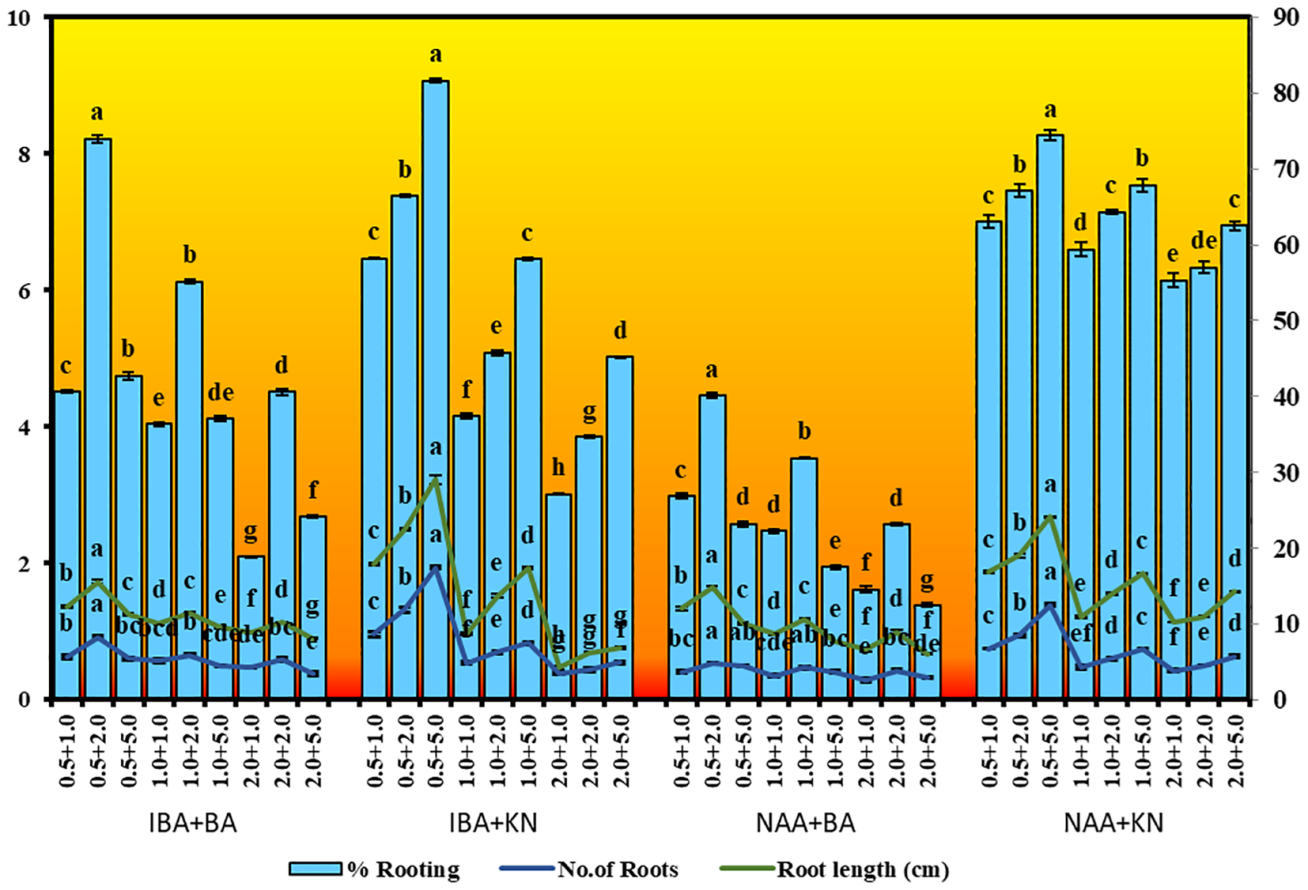
Root inductions in *W. chinensis* through nodal segments after 12 weeks in the presence of different hormone combination. Different lowercase letters are representing significance at p = 0.05 according to Duncan’s multiple range test.

**Figure 4:**
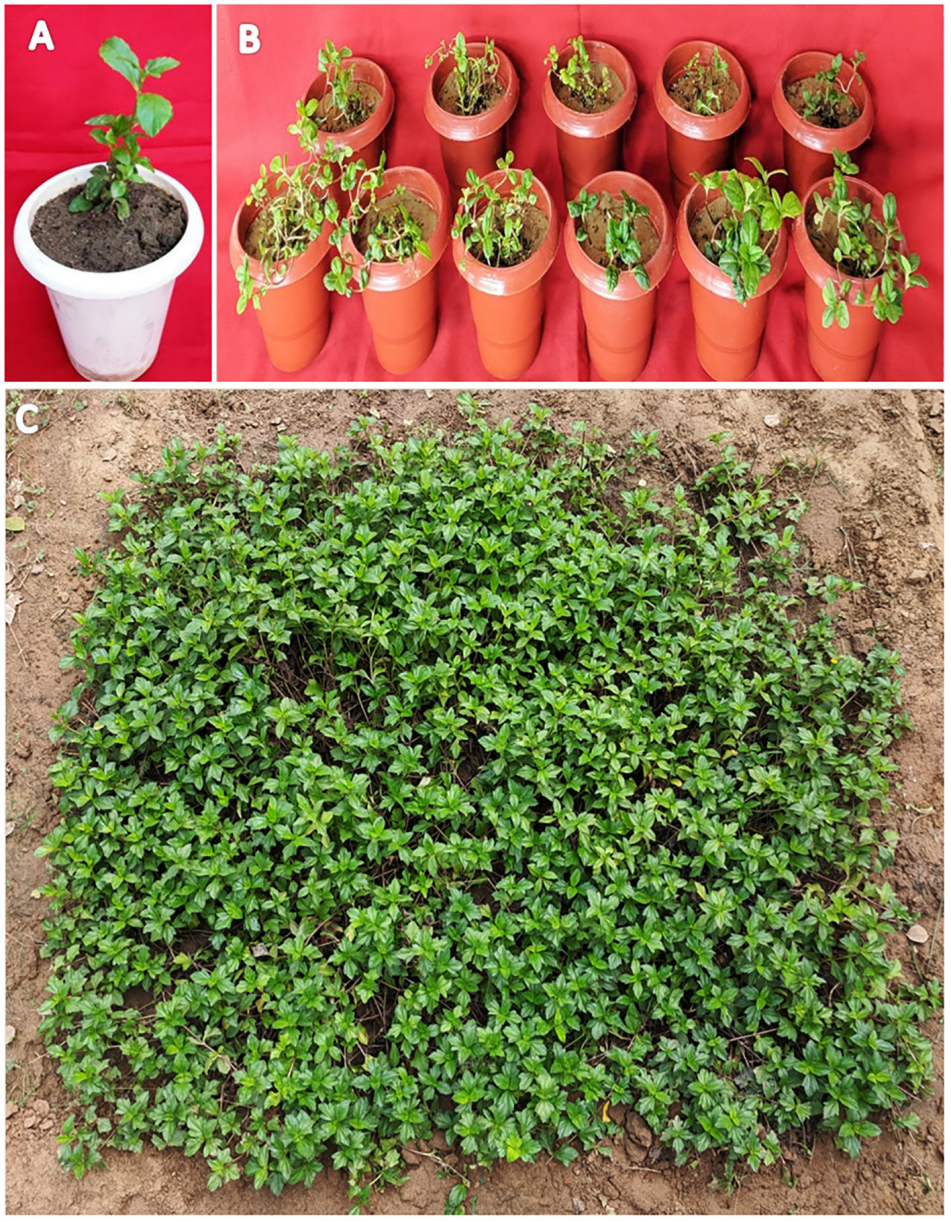
Transplantation of in vitro grown *W. chinensis* **(A)**: Transfer of plants in plastic pots containing a mixture of soilrite and soil (1:1), **(B)**: Regenerants transferred to greenhouse after successful acclimatization for hardening, **(C)**: 6-month-old regenerants grown under natural conditions.

*In vitro*-regenerated 4.0–5.0-cm shoots rooted on all media containing auxins and cytokinins (**Table 2**). Here, 74% rooting was observed in cultures grown in the presence of MS medium containing IBA (0.5 mg/L) along with BA (2.0 mg/L) within 12 weeks and 40.17% rooting in the presence of NAA (0.5 mg/L) + BA (2.0 mg/L)-containing medium. A higher concentration of KN (5.0 mg/L) with NAA (0.5 mg/L) resulted in 74.43% rooting with 1.39 roots of 2.68 cm after 12 weeks. Of all the concentrations and combinations of growth regulators tried, MS medium containing IBA (0.5 mg/L) and KN (5.0 mg/L) was found to be best (**Figure 3**). The shoots showed 81.67% rooting efficacy with an average of 1.94 roots of 3.23 cm within 12 weeks. However, the rooted plantlets when subcultured on MS liquid medium containing IBA + KN (0.5 + 5.0) resulted in approximately a 2-fold increase in root (4.5 roots/shoot) as well as a 7-fold increase in root length (22 cm) as compared with roots on semisolid same growth regulator containing medium even in 4 weeks (**Figures 1E, F**). The hardened plants transferred to the field showed 92% survival rate and grew normally in outdoor conditions (**Figures 4A–C**).

**Table 2:** Effect of plant growth regulators on root induction in *W. chinensis* through nodal segments after 12 weeks.

MS semisolid + PGR (mg/l)	Percent root induction	Average no. of roots per shoot	Average length of roots (in cm)
IBA + BA			
0.5+1.0	40.67 ± 0.152^c^	0.63 ± 0.021^b^	1.37 ± 0.021^b^
0.5+2.0	74.00 ± 0.525^a^	0.92 ± 0.017^a^	1.73 ± 0.022^a^
0.5+5.0	42.73 ± 0.472^b^	0.60 ± 0.025^bc^	1.25 ± 0.014^c^
1.0+1.0	36.33 ± 0.237^e^	0.57 ± 0.028^bcd^	1.12 ± 0.018^d^
1.0+2.0	55.17 ± 0.260^b^	0.65 ± 0.019^b^	1.27 ± 0.012^c^
1.0+5.0	37.07 ± 0.321^de^	0.49 ± 0.010^cde^	1.06 ± 0.014^e^
2.0+1.0	18.83 ± 0.115^g^	0.47 ± 0.006^de^	0.99 ± 0.021^f^
2.0+2.0	40.63 ± 0.412^d^	0.59 ± 0.026^bc^	1.13 ± 0.017^d^
2.0+5.0	24.17 ± 0.144^f^	0.38 ± 0.026^e^	0.90 ± 0.011^g^
IBA+KN			
0.5+1.0	58.22 ± 0.091^c^	0.96 ± 0.0319^c^	1.99 ± 0.0213^c^
0.5+2.0	66.44 ± 0.181^b^	1.32 ± 0.0327^b^	2.50 ± 0.0223^b^
0.5+5.0	81.67 ± 0.272^a^	1.94 ± 0.0236^a^	3.23 ± 0.0650^a^
1.0+1.0	37.44 ± 0.362^f^	0.54 ± 0.0141^f^	0.97 ± 0.0119^f^
1.0+2.0	45.80 ± 0.340^e^	0.70 ± 0.0170^e^	1.52 ± 0.0262^e^
1.0+5.0	58.13 ± 0.109^c^	0.83 ± 0.0223^d^	1.93 ± 0.0241^d^
2.0+1.0	27.11 ± 0.090^h^	0.39 ± 0.0218^g^	0.47 ± 0.0272^h^
2.0+2.0	34.70 ± 0.242^g^	0.44 ± 0.0236^g^	0.69 ± 0.0094^g^
2.0+5.0	45.17 ± 0.136^d^	0.55 ± 0.0177^f^	0.76 ± 0.0189^g^
NAA+BA			
0.5+1.0	26.87 ± 0.381^c^	0.41 ± 0.012^bc^	1.34 ± 0.028^b^
0.5+2.0	40.17 ± 0.360^a^	0.53 ± 0.015^a^	1.64 ± 0.026^a^
0.5+5.0	23.22 ± 0.327^d^	0.50 ± 0.007^ab^	1.12 ± 0.019^c^
1.0+1.0	22.22 ± 0.181^d^	0.35 ± 0.017^cde^	0.96 ± 0.017^d^
1.0+2.0	31.89 ± 0.093^b^	0.47 ± 0.020^ab^	1.18 ± 0.017^c^
1.0+5.0	17.48 ± 0.236^e^	0.41 ± 0.019^bc^	0.85 ± 0.014^e^
2.0+1.0	14.55 ± 0.367^f^	0.29 ± 0.022^e^	0.74 ± 0.028^f^
2.0+2.0	23.18 ± 0.150^d^	0.43 ± 0.026^bc^	0.99 ± 0.027^d^
2.0+5.0	12.48 ± 0.236^g^	0.33 ± 0.012^de^	0.67 ± 0.009^f^
NAA+KN			
0.5+1.0	63.10 ± 0.860^c^	0.75 ± 0.0027^c^	1.87 ± 0.0170^c^
0.5+2.0	67.17 ± 0.792^b^	0.94 ± 0.0227^b^	2.11 ± 0.0223^b^
0.5+5.0	74.43 ± 0.644^a^	1.39 ± 0.0247^a^	2.68 ± 0.0141^a^
1.0+1.0	59.40 ± 0.984^d^	0.48 ± 0.0258^ef^	1.22 ± 0.0166^e^
1.0+2.0	64.33 ± 0.272^c^	0.60 ± 0.0100^d^	1.56 ± 0.0166^d^
1.0+5.0	67.87 ± 0.891^b^	0.74 ± 0.0170^c^	1.85 ± 0.0141^c^
2.0+1.0	55.30 ± 0.953^e^	0.43 ± 0.0118^f^	1.14 ± 0.0027^f^
2.0+2.0	57.03 ± 0.830^de^	0.50 ± 0.0027^e^	1.22 ± 0.0119^e^
2.0+5.0	62.50 ± 0.634^c^	0.63 ± 0.0196^d^	1.59 ± 0.0098^d^

Data in each column represents mean ± SE followed by different letters that are significantly different at p = 0.05 according to Duncan’s multiple range test.

### 3.3 Extraction and quantification of wedelolactone

**Figure 5:**
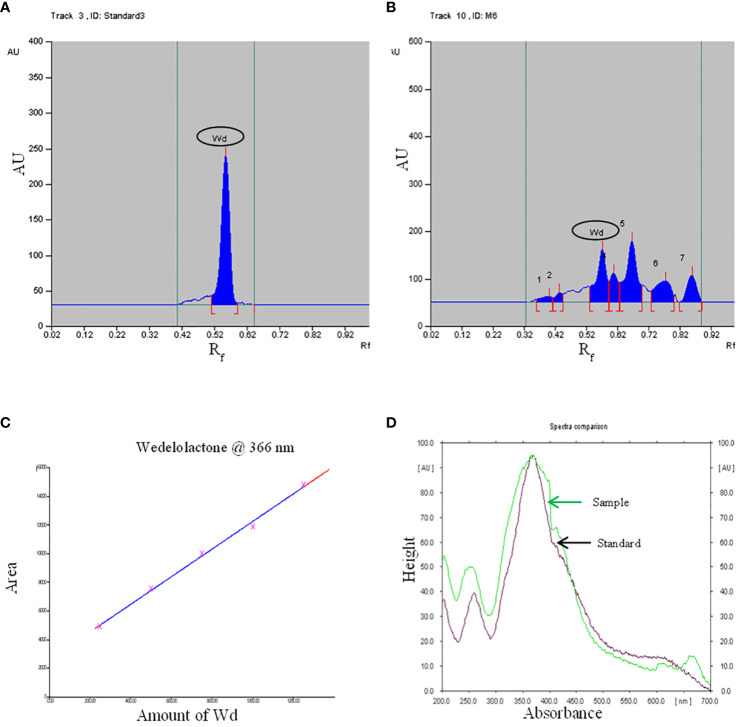
HPTLC chromatogram - **(A)**: Standard, **(B)**: Sample, **(C)**: Standard curve, **(D)**: Spectral comparison of Wd. (Here Wd - Wedelolactone).

Growth hormone-enriched MS medium was also favorable for enhancement in the yield of wedelolactone in *W. chinensis*. For standardization of the solvent system, a standard of wedelolactone was used. Out of the polar and nonpolar solvents used, toluene:ethyl acetate:formic acid (5:4:1) was found to be best for the detection and quantification of wedelolactone with an R_f_ value of 0.56 (**Figures 5A, B**). The maximum wavelength at which wedelolactone absorbs the maximum light is 366 nm.

**Figure 6:**
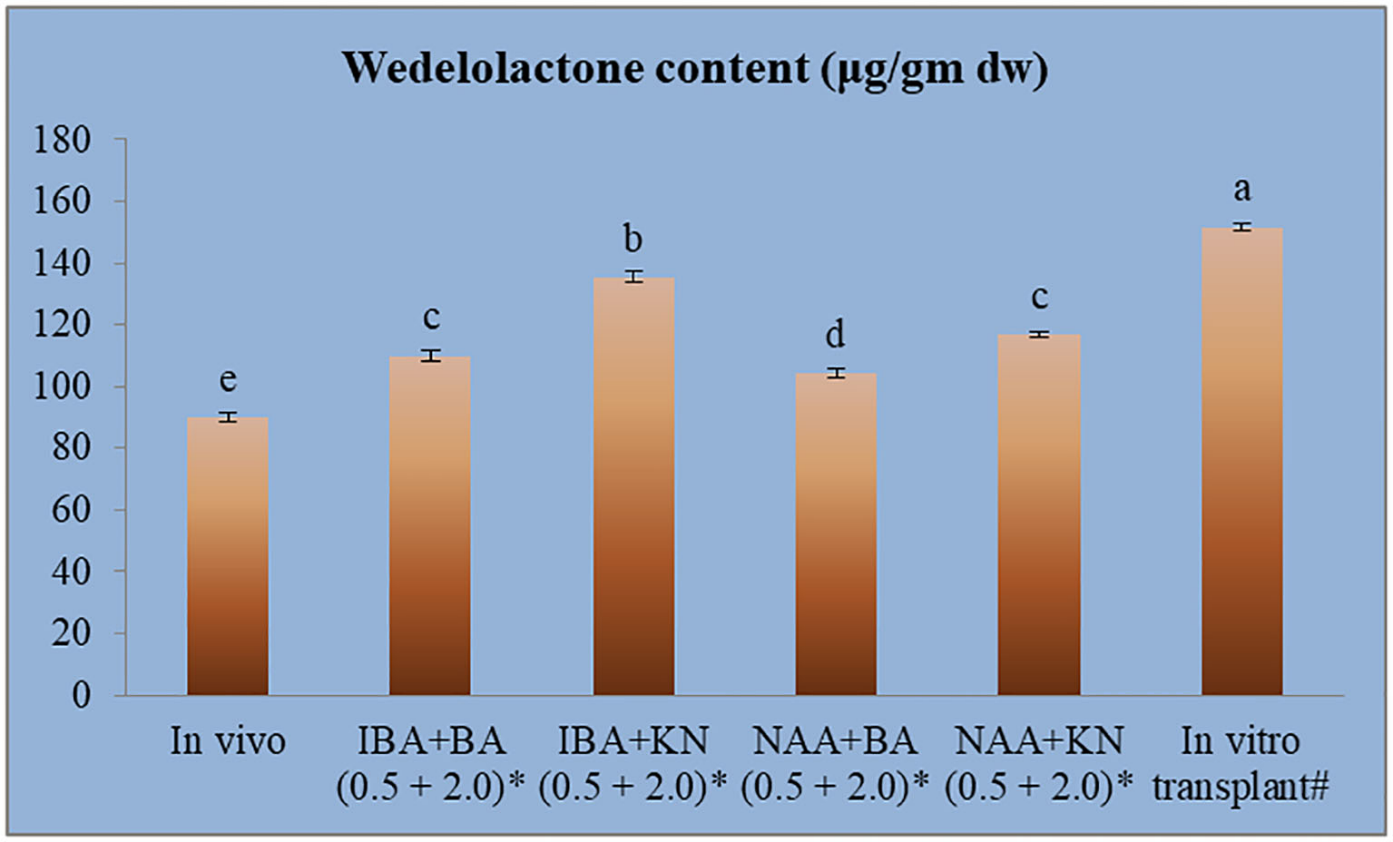
Quantative estimantion of wedelolactone in *in vivo* and *in vitro* grown plantlets grown under influence of different growth regulators. *4-month-old Plantlet raised on MS + (mg/l), #14-month-old in vitro raised plantlet transferred to the field (total age: 6 months). Different lowercase letters are representing significance at p = 0.05 according to Duncan’s multiple range test.

The calibration graph was constructed by plotting the peak area vs. the amount of wedelolactone loaded over a range of 50–250 ng (**Figure 5C**). The standard curve was achieved with a regression value of 0.99. Wedelolactone was estimated in the samples based on the R_f_ value (0.56) of the standard compound. The concentration of wedelolactone was calculated from the formula mentioned in the *Materials and Methods*. Furthermore, for confirmation, spectral comparison was done. The peak of sample overlapping with the wedelolactone standard is authenticating the results (**Figure 5D**). The wedelolactone content in *in vivo*-grown plants was detected at 89.95 μg/g dw. In *in vitro*-grown plants raised on the best regeneration medium (MS supplemented with 0.5 mg/L IBA and 5.0 mg/L KN), 1.50-fold enhancement in wedelolactone content (135.45 μg/g dw) was detected (**Figure 6**). The plantlets raised on this medium upon transplantation showed more accumulation of wedelolactone as compared with field-grown plants (**Table 3**).

**Table 3:** Effect of plant growth regulators and culture duration on wedelolactone content in *W. chinensis*.

S.No.	Plant source	Wd content (In μg/g dw)
1	*In vivo* grown plant	89.95 ± 1.29^e^
2	IBA+BA (0.5 + 2.0)^*^	110.08 ± 1.65^c^
3	IBA+KN (0.5 + 5.0)^*^	135.45 ± 1.72^b^
4	NAA+BA (0.5 + 2.0)^*^	104.27 ± 1.52^d^
5	NAA+KN (0.5 + 5.0)^*^	116.89 ± 1.01^c^
6	Transplanted plantlet^#^	151.71 ± 1.28^a^

Data in each column represents mean ± SE.

^*^4-month-old Plantlet raised on MS + (mg/l), ^#^14-month-old in vitro raised plantlet transferred to the field (total age: 6 months).

### 3.4 Genome size and genetic stability

**Figure 7:**
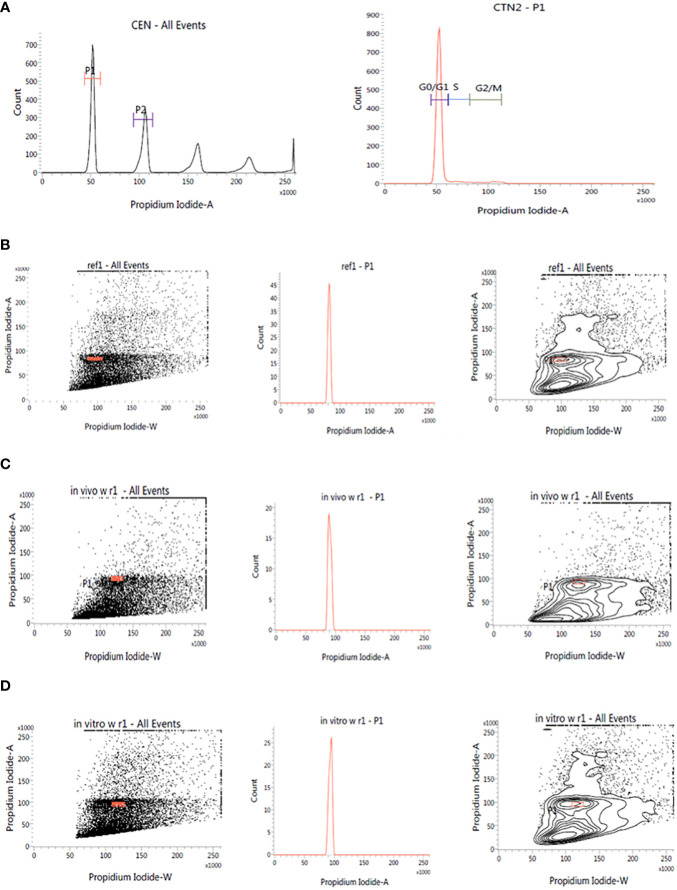
Estimation of nuclear DNA content in absolute units. **(A)**: Histogram of CEN (Chicken erythrocyte nuclei) and CTN (Calf thymocyte nuclei). The dot plot, histogram, and contour of **(B)**: P. pinnata (Standard), **(C)**: in vivo grown *W. chinensis*, **(D)**: 6-month-old transplanted plants of *W. chinensis* obtained through direct organogenesis.

This investigation also evaluated the influence of *in vitro* culture conditions on the genetic stability of *W. chinensis*. The 2C DNA content of 4-month-old regenerants raised via direct organogenesis, 6-month-old transplanted *W. chinensis* was compared with that of field-grown plants. Singlet cell population of standard (*P. pinnata*) and field- and *in vitro*-grown *W. chinensis* was gated at 100 position on (PI-A vs. count) histogram, confirming the correct gating. The discrete populations of cells are also easily visualized on contour maps, further confirming the results (**Figures 7A–D**). The Coefficient of variation (CV) of the histogram peaks of standard and test samples is below 3%, indicating the data with better resolution, as both are inversely correlated. The 2C DNA content of field-grown plants of *W. chinensis* was 2.80 pg. *In vitro*-raised 4-month-old plantlets and 6-month-old transplanted plants showed 2.87 and 2.86 pg of 2C DNA content, respectively, conforming similarity in genome size to field-grown *W. chinensis* (**Table 4**). Therefore, no major alteration in genome size in *in vitro*-regenerated plants was noted when compared with *in vivo*-grown plants. Thus, the genetic integrity of the regenerated plants is maintained during *in vitro* conditions as well as genome is stable even after hardening and transplantation to field conditions.

**Table 4:** 2C DNA content and Coefficient of variation in *W. chinensis, in vivo* grown plant, tissue culture regenerated 4-month-old plantlets and 6-month-old transplanted plants in the field.

Plant source	2C DNA content	Coefficient of variance (CV)
*In vivo* grown	2.80 ± 0.022	2.07
4-month-old plantlets	2.87 ± 0.008	2.70
6-month-old plantlets	2.86 ± 0.011	2.63

Data in each column represents mean ± SE.

### 3.5 Effect of CuSO_4_ on *in vitro*-raised plantlets

*In vitro*-raised shoots were cut into 4–5-cm-long pieces and transferred to a regeneration medium that is MS + IBA (0.5 mg/L) + KN (5.0 mg/L) with different concentrations of CuSO_4_ (0–125 μM).

#### 3.5.1 Effect of CuSO_4_ on shoot fresh and dry weight

**Figure 8:**
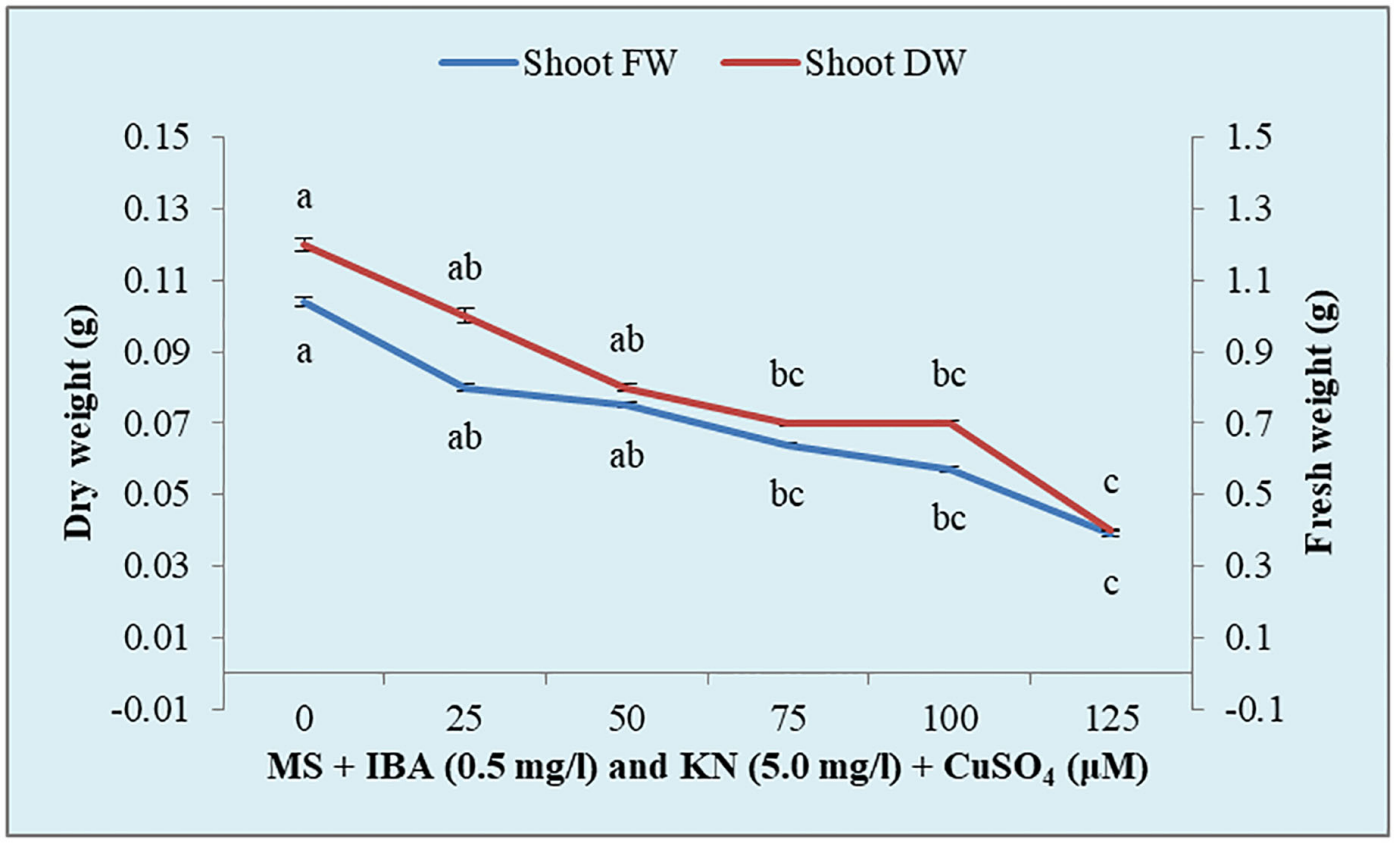
Effect of CuSO_4_ on shoot fresh and dry weight in 12-week-old *in vitro* raised plantlets of *W. chinensis*. Different lowercase letters are representing significance at p = 0.05 according to Duncan’s multiple range test.

There was a significant variation in the fresh weight of shoots among different concentrations of CuSO_4_ used. CuSO_4_ treatment significantly reduced the fresh weight in all of the shoots. The fresh weight varied from 0.39 g to 0.80 g (**Figure 8**). Similarly, the shoot dry weight was reduced from 0.10 g (25 μM) to 0.04 g (125 μM) by increasing the concentration of CuSO_4_ in the medium (**Table 5**).

**Table 5:** Shoot fresh and dry weight in 12-week-old *in vitro* raised plantlets of *W. chinensis* grown in the presence of CuSO_4_.

Shoots grown on MS + IBA (0.5 mg/l) and KN (5.0 mg/l) + CuSO_4_ (μM)	Shoot fresh weight (g)	Shoot dry weight (g)
00	1.04 ± 0.135^a^	0.12 ± 0.019^a^
25	0.80 ± 0.093^ab^	0.10 ± 0.021^ab^
50	0.75 ± 0.068^ab^	0.08 ± 0.009^ab^
75	0.64 ± 0.051^bc^	0.07 ± 0.006^bc^
100	0.57 ± 0.093^bc^	0.07 ± 0.006^bc^
125	0.39 ± 0.058^c^	0.04 ± 0.002^c^

The data were statistically analyzed using Duncan’s multiple range test. Values are mean ± SE. In the same column, significant differences at the P≤0.5 level are indicated by different letters.

#### 3.5.2 Effect of CuSO_4_ on total protein content

**Figure 9:**
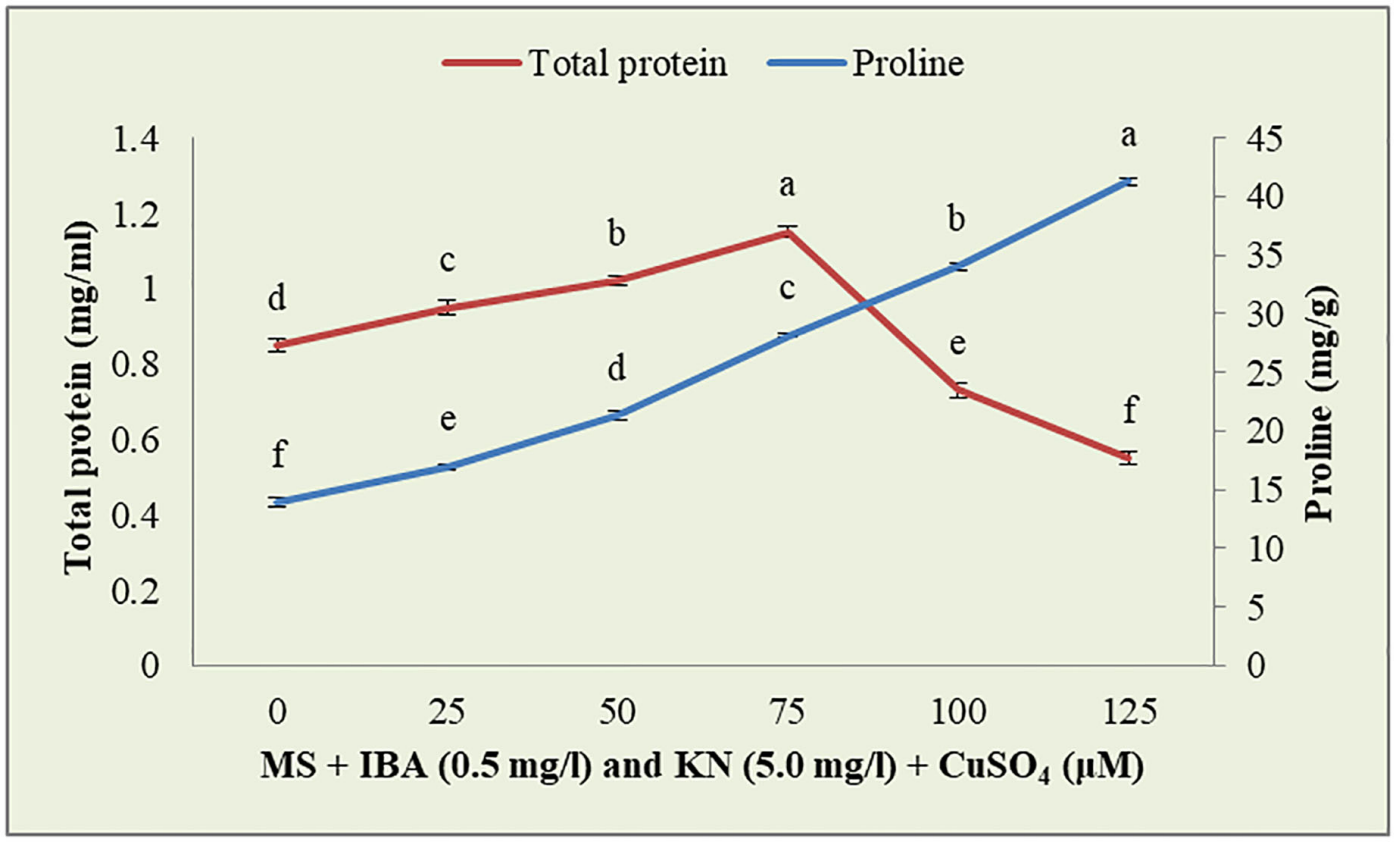
Effect of CuSO_4_ on total protein and proline content in 12-week-old *in vitro* raised plantlets of *W. chinensis*. Different lowercase letters are representing significance at p = 0.05 according to Duncan’s multiple range test.

CuSO_4_ caused an enhancement in the total protein content from 0.95 to 1.15 g^-1^ fw until 75 μM (**Figure 9**). On increasing the CuSO_4_ concentration from 75 μM to 100 μM and 125 μM, protein content reduced significantly (**Table 6**).

**Table 6:** Total protein content in 12-week-old *in vitro* raised plantlets of *W. chinensis* grown in the presence of CuSO_4_.

Shoots grown on MS + IBA (0.5 mg/l) and KN (5.0 mg/l) + CuSO_4_ (μM)	Total protein content (mg/ml)
00	0.85 ± 0.017^d^
25	0.95 ± 0.019^c^
50	1.02 ± 0.012^b^
75	1.15 ± 0.014^a^
100	0.73 ± 0.020^e^
125	0.55 ± 0.017^f^

The data were statistically analyzed using Duncan’s multiple range test. Values are mean ± SE. In the same column, significant differences at the P≤0.5 level are indicated by different letters.

#### 3.5.3 Effect of CuSO_4_ on proline content

Enhanced proline content was observed in *in vitro*-raised shoots grown on different concentrations of CuSO_4_. In the control, shoots showed 13.88 mg/g fw proline content. On increasing the concentration of CuSO_4_ in the medium from 25 μM to 125 μM, proline content increased significantly (**Figure 9**). Shoots produced maximum proline (41.27 mg/g) at 126 μM and minimum (16.96 mg/g) at 25-μM CuSO_4_ concentrations (**Table 7**).

**Table 7:** Proline content in 12-week-old *in vitro* raised plantlets of *W. chinensis* grown in the presence of CuSO_4_.

Shoots grown on MS + IBA (0.5 mg/l) and KN (5.0 mg/l) + CuSO_4_ (μM)	Proline content (mg/g fw)
00	13.88 ± 0.396^f^
25	16.96 ± 0.231^e^
50	21.30 ± 0.427^d^
75	28.18 ± 0.107^c^
100	34.02 ± 0.345^b^
125	41.27 ± 0.381^a^

The data were statistically analyzed using Duncan’s multiple range test. Values are mean ± SE. In the same column, significant differences at the P≤0.5 level are indicated by different letters.

#### 3.5.4 Effect of CuSO_4_ on chlorophyll and carotenoid content

Chlorophyll and carotenoid contents are also affected by the presence of CuSO_4_ in the medium. Shoots grown on control medium showed 1.93 mg/g fw and 0.46 µg/g fw of chlorophyll and carotenoid, respectively. The content of both chlorophyll and carotenoid was enhanced by the effect of CuSO_4_ and found maximum at 75 μM concentration. After increasing the concentration of CuSO_4_ in the medium, chlorophyll and carotenoid contents declined. The maximum level of chlorophyll and carotenoid content was observed on medium containing 75 μM of CuSO_4_ (**Table 8**).

**Table 8:** Chlorophyll and carotenoid content in 12-week-old *in vitro* raised plantlets of *W. chinensis* grown in the presence of CuSO_4_.

Shoots grown on MS + IBA (0.5 mg/l) and KN (5.0 mg/l) + CuSO_4_ (μM)	Chlorophyll A content (mg/g fw)	Chlorophyll B content (mg/g fw)	Total Chlorophyll content (mg/g fw)	Carotenoid content (µg/g fw)
00	1.48 ± 0.009^d^	0.44 ± 0.027^d^	1.93 ± 0.034^d^	0.46 ± 0.014^d^
25	1.66 ± 0.020^c^	0.62 ± 0.036^c^	2.28 ± 0.044^c^	0.55 ± 0.008^c^
50	1.75 ± 0.021^b^	0.78 ± 0.034^b^	2.53 ± 0.054^b^	0.63 ± 0.008^b^
75	2.18 ± 0.019^a^	1.28 ± 0.033^a^	3.46 ± 0.016^a^	0.70 ± 0.034^a^
100	1.14 ± 0.019^e^	0.34 ± 0.020^de^	1.48 ± 0.019^e^	0.39 ± 0.011^d^
125	0.55 ± 0.025^f^	0.26 ± 0.044^e^	0.82 ± 0.060^f^	0.22 ± 0.016^e^

The data were statistically analyzed using Duncan’s multiple range test. Values are mean ± SE. In the same column, significant differences at the P≤0.5 level are indicated by different letters.

#### 3.5.5 Effect of CuSO_4_ on wedelolactone content

**Figure 10:**
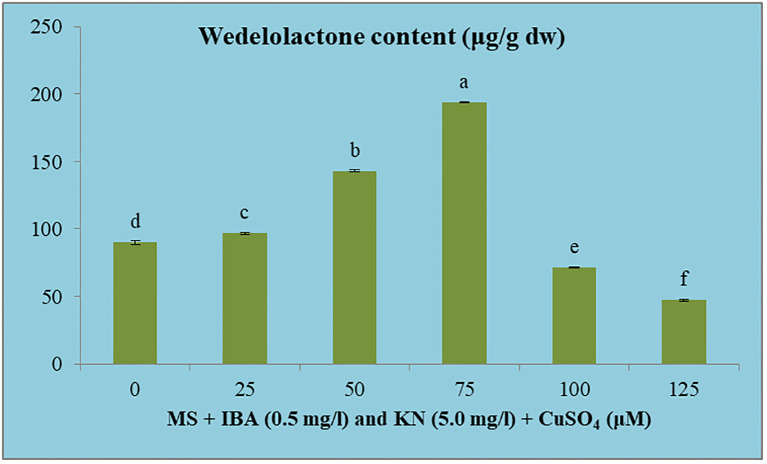
Effect of CuSO_4_ on wedelolactone content in 12-week-old in vitro raised plantlets of *W. chinensis*. Different lowercase letters are representing significance at p = 0.05 according to Duncan’s multiple range test.

The wedelolactone content extracted from the shoots grown on different concentrations of CuSO_4_ containing regeneration medium showed variation. Maximum wedelolactone was quantified at 75 μM (193.90 μg/g dw) and minimum (47.13 μg/g dw) was found at 125 μM CuSO_4_ (**Figure 10**). Here, 116% enhancement in wedelolactone content was detected in regenerated shoots grown on MS regenerated medium containing 75 μM CuSO_4_ as compared to *in vivo*-grown plants (**Table 9**).

**Table 9:** Wedelolactone content in 12-week-old *in vitro* raised plantlets of *W. chinensis* grown in the presence of CuSO_4_.

Shoots grown on MS + IBA (0.5 mg/l) and KN (5.0 mg/l) + CuSO_4_ (μM)	Wedelolactone content (μg/g dw)
00	89.95 ± 1.29^d^
25	96.70 ± 0.74^c^
50	142.99 ± 0.63^b^
75	193.90 ± 0.58^a^
100	71.43 ± 0.55^e^
125	47.13 ± 0.96^f^

The data were statistically analyzed using Duncan’s multiple range test. Values are mean ± SE. In the same column, significant differences at the P≤0.5 level are indicated by different letters.

## 4 Discussion

With the prevalence of cancer escalating worldwide, the demand for potent and effective anticancer agents is more pressing than ever. *W. chinensis*, housing the bioactive compound wedelolactone, presents a unique opportunity for therapeutic exploration. Our study’s innovative approach, encompassing *in vitro* rapid multiplication and heightened wedelolactone content, addresses crucial gaps in both conservation and production of this medicinal plant. By offering a sustainable propagation method and amplifying the yield of therapeutically significant molecules, we contribute to the advancement of therapeutic research, potentially leading to groundbreaking discoveries in cancer therapy and other medical applications.

In the present study, the nodal segments are used as an explant for direct regeneration from axillary buds to obtain true-to-type plants. Previously, *in vitro* clonal propagation of *W. chinensis* was done by Agarwala et al. (2010) and Rahman and Bhadra (2011) using nodes as explants with 70% and 80% survival rates, respectively (Agarwala et al., 2010; Rahman and Bhadra, 2011). Whereas Tsai and To (2021) determined that stems and mature leaves of *W. chinensis* are not suitable materials for plant regeneration. They used only the youngest two leaves and successfully regenerated *W. chinensis *via indirect organogenesis. Maximum of 23% shoot regeneration was found on MS medium supplemented with NAA (0.5 mg/L), Thidiazuron (TDZ) (0.75 mg/L), GA_3_ (1 mg/L), adenine (3.75 mg/L), 3% sucrose, and 0.8% agar at pH 5.8. The *in vitro*-grown shoots induced rooting on MS medium containing NAA (3 mg/L) and 3% sucrose with or without 0.8% agar and were successfully transferred to soil (Tsai and To, 2021).

In the present study, both the cytokinins (KN and BA) in combination with lower concentrations of IBA and NAA facilitated shoot induction from axillary buds. Although from all combinations used, MS medium augmented with KN at 5.0 mg/L along with IBA 0.5 mg/L proved to be the most effective for shoot regeneration. Similar results were also obtained in cucumber (Abu-Romman et al., 2015). On the other hand, the effectiveness of BA over KN for shoot regeneration has been earlier documented in *Eclipta alba* (Yesmin et al., 2016) and *Gymnema sylvestre* (Isah, 2019). The effective role of adenine sulfate at a higher concentration (25 mg/L) in the culture medium improves the frequency of shoot development and helps in the recovery of the leaves from chlorotic symptom in *W. chinensis* (Tsai and To, 2021).

For every micropropagation protocol, successful rooting of regenerated shoots is essential for establishment in the soil as well as survival on *in vitro*-raised plants in field conditions. *In vitro*-raised shoots gave rooting on all combinations of auxins and cytokinins. The best rooting response was observed on MS supplemented with IBA (0.5 mg/L) along with KN (5.0 mg/L). A similar response was observed by Dhaka and Kothari (2005) in *E. alba*. IBA is the most commonly used auxin for root induction not only in herbal plants but also in woody trees (Goyal et al., 2012). Root induction and proliferation on IBA-enriched MS medium were also observed by several workers in various medicinal plants, e.g., *E. alba* (Sharma et al., 2013), *Pistacia vera* (Tilkat et al., 2009), *Pentanema indicum* (Sivanesan and Jeong, 2007), and *Azadiracha indica* (Shahin-uz-zaman et al., 2008). Divergent to this, other auxins have been reported for rooting in *Thymus vulgaris* (Ozudogru et al., 2011). At higher concentrations of IBA, the percentage of rooting decreased and callus formation occurred at the basal cut end as also reported in *Pithecellobium dulce* (Goyal et al., 2012). The 4-month-old rooted plants were sequentially hardened and successfully transplanted into the field. The plantlets showed 92% survival and attained maturity and flowered. A similar approach was also reported earlier in *E. alba* (Sharma et al., 2013).

The use of MS medium (devoid of agar) has significantly improved micropropagation efficiency and production of healthy plantlets in several plant species and is an attractive alternative to growing cultures on a semisolid medium (Vaidya et al., 2019). However, rapid multiplication under *in vitro* conditions on liquid culture was reported in the roots of *Cleome rosea* (da Silva Cordeiro et al., 2015). Currently, there are no studies that have explored the use of liquid culture systems for the micropropagation of *W. chinensis*. In the present study, higher number and length of roots differentiated in liquid MS medium augmented with IBA (0.5 mg/L) and KN (5.0 mg/L) within 4 weeks. Improved aeration and water availability in the culture resulted in an improved root proliferation rate in the liquid medium. Similarly, it is also reported in a variety of plant species (Adelberg and Cousins, 2006; Tascan et al., 2010; Croom et al., 2016). Liquid media produced the highest shoot length and rooting percentage in *Boswellia serrata* (Suthar et al., 2011) and *Typhonium flagelliforme* (Rezali et al., 2017). Despite the benefits of the liquid culture system, *Scutellaria* species that are not suitable for liquid culture led to the development of hyperhydricity (Tascan et al., 2010).

Plant tissue culture technique led to enhancement in the secondary metabolite level over the field-grown plants (Naik and Al-Khayri, 2016). Various factors like growth regulators, carbon source, temperature, and photoperiod affect the biosynthetic pathway of secondary metabolites (Jayathirtha and Mishra, 2003). In the present study, 116% increase in wedelolactone content was achieved in *in vitro*-grown plantlets under Cu stress as compared with plants grown under natural conditions. Enhanced (2.7–6.4-fold) wedelolactone content was previously recorded in the regenerated plants of *E. alba* as compared with the *in vivo*-grown plants (Zafar and Sagar, 1999).

Flow cytometry based on the principle of DNA-selective fluorochromes is now the prevailing method for the measurement of nuclear DNA content in plants. Ease of sample preparation and high sample throughput make it generally better suited than other methods such as RAPD to estimate genome size and level of generative polyploidy (Doležel et al., 2007). Earlier, Tsai et al. (2021) successfully examined polyploidy analysis in *in vitro*-grown *W. chinensis* using a flow cytometer (Tsai et al., 2021).

The present report validates genetic uniformity of *in vivo*-grown and *in vitro*-regenerated plants of *W. chinensis* by using a flow cytometer. The peaks of regenerated plants are similar to the peak of the 2C DNA level of *in vivo*-grown plants. This confirms that the genetic fidelity among regenerated plants of *W. chinensis* was unaltered and maintained even after successful transplantation in the field. The present study is in close agreement with that in micropropagated *Carum copticum* (Niazian et al., 2017) and *Rauvolfia serpentina* (Zafar et al., 2019) where genetic stability was proved using flow cytometry. Similarly, genome size stability has been reported in *in vitro*-regenerated plants of *Coriandrum sativum* (Ali et al., 2017) and *Gladiolus* (Mujib et al., 2017). In contrast to the present study, lower nuclear DNA content was obtained in *in vitro*-regenerated plants of *Pueraria lobata* than that in the control seedlings (Makowczyńska et al., 2008). Some gross genome variability has also been noted in tissue culture-regenerated plants of *Nitraria tangutorum* (Yang et al., 2010) and oil palm (Lucia et al., 2011).

Soil contamination with heavy metals is a widespread environmental issue, originating from urbanization, industrial growth, mining activities, municipal waste, and agriculture practices (Masindi and Muedi, 2018; Zwolak et al., 2019). Cu is an essential micronutrient for plants. It contributes to different physiological processes, including photosynthetic electron transport, mitochondrial respiration, cell wall metabolism, hormone signaling, DNA transcription, protein trafficking, and protein regulation (Jung, 2008; Ullah et al., 2020). However, an extreme amount makes it toxic for plants because of its redox properties. Excess Cu affects photosynthetic and respiratory processes, inhibits plant growth, decreases nutrient uptake, and produces reactive oxygen species (Ravet and Pilon, 2013; Zandi et al., 2020).

In the present investigation, stress tolerance of the regenerants was tested under different concentrations of CuSO_4_. The shoot fresh and dry weights were reduced by increasing the concentration of CuSO_4_ in the medium. However, enhanced shoot biomass accumulation is reported in *Tagetes minuta* in the presence of lead (del Carman Sosa et al., 2016). Whereas total protein, proline as well as chlorophyll, and carotenoid contents were increased up to 75 μM and after that decreased on further increase in CuSO_4_. Similarly, increased chlorophyll and carotenoid contents were observed in *T. minuta* in the presence of lead (del Carman Sosa et al., 2016) and an enhanced level of proline content in *Cajanus cajan* due to Cu stress (Hayat et al., 2021).

Heavy metals act as abiotic stress agents, causing oxidative damage to the plant. Plants can recognize these threat signals and activate various defense responses. Biosynthesis and accumulation of secondary metabolites are vital detoxification mechanisms that help to relieve the detrimental effects caused by toxic heavy metals (Anjitha et al., 2021). In the present study, wedelolactone content was enhanced from 0 to 75 μM CuSO_4_ concentrations, and after that, further increasing CuSO_4_ reported reduced yield. A maximum 100% increase in wedelolactone content was detected at 75 μM CuSO_4_ concentration compared to *in vivo* plants. Various researchers obtained enhanced secondary metabolite under Cu stress in different plants, such as enhanced diosgenin yield in *Dioscorea bulbifera* (Narula et al., 2005), quercetin content in *Pluchea lanceolata* (Kumar et al., 2004), saponin in *Bacopa* (Srivastava et al., 2002), and phytochelatins in Antarctic *Colobanthus* (Contreras et al., 2018) and increased phenylpropanoid biosynthesis in the adventitious root culture of *Althaea officinalis* (Park et al., 2021).

## 5 Conclusion

The present research emphasized a well-organized, low-cost, and rapid *in vitro* mass multiplication protocol for the conservation of less exploited medicinally important *W. chinensis*. The remarkability of this protocol is based on an effective sterilization procedure followed by the use of plant growth regulators to promote increased multiplication rates in a short duration under *in vitro* conditions. Additionally, the protocol highlights the acclimatization of *in vitro*-grown plantlets to natural conditions with high efficiency and survival rates for the conservation of this plant. Another application of plant tissue culture is the production of true-to-type plants for usefulness on an industrial scale for the production of secondary metabolites. Keeping this in mind instead of using RAPD that gives low reproducibility, we are the first to report the use of flow cytometry, a flourochromatic based advanced and accurate method, for checking the genetic uniformity of tissue culture-raised *W. chinensis*. The regenerated plants obtained through direct regeneration were genetically stable even after transplantation, and their genetic profiling was similar to field-grown plants. CuSO_4_ in the regeneration medium not only enhances stress tolerance but also leads to an increase in the production of secondary metabolites. Enhanced levels of wedelolactone obtained in this study can be exploited for the treatment of different types of cancer.

## Data availability statement

The original contributions presented in the study are included in the article/supplementary material. Further inquiries can be directed to the corresponding authors.

## Author contributions

RS: Data curation, Formal analysis, Funding acquisition, Methodology, Project administration, Resources, Validation, Visualization, Writing – original draft, Writing – review & editing. SN: Software, Supervision, Writing – review & editing. AN: Supervision, Writing – review & editing. HF: Supervision, Writing – review & editing.
