# Supplementary figures and images for: Enhanced wedelolactone content in in vitro-raised genetically uniform Wedelia chinensis under the influence of CuSO4
Source: Front Plant Sci. 2023 Dec 19;14:1281445. doi: 10.3389/fpls.2023.1281445 (PMC10758438; doi:10.3389/fpls.2023.1281445)

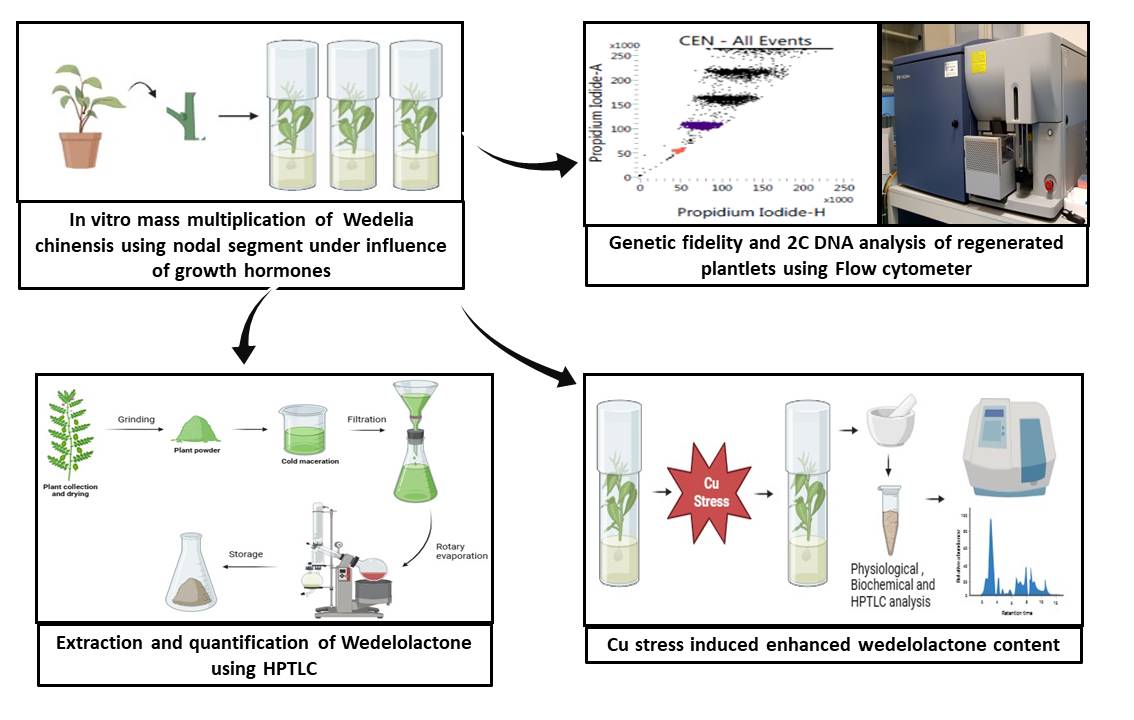

Supplement: Supplementary file 1 [file Image_1.jpeg]
